# Observations on Tuberculous Consumption, &c. &c.

**Published:** 1842-01-01

**Authors:** 


### Observations on Tuberculous Consumption, &c. &c. By *J. S. Campbell*, M.D. Senior Physician to the Marylebone General Dispensary, &c. 8vo. pp. 402. Bailliere, Oct. 1841.

This is a most laboured essay, more than half of which (221 pages) is taken up with recondite disquisitions on life, necessity, vital actions, tubercles, humoral pathology, inflammation, &c. &c. in which the author has evinced abundance of ingenuity, research, and talent for investigation.

We must, however, pass over the first part of the work, merely exhibiting the conclusions which our author has drawn from the matters contained therein.

"In concluding this chapter it may be desirable to take a rapid glance at the points which it has been my endeavour to establish in the preceding pages. I have considered tubercular consumption not as a simple but compound affection, essentially consisting of distinct stages, and distinct varieties of morbid action, some of which are referrible to the organ immediately involved, and some to distant parts, their morbid influences being reciprocally propagated through the medium of the blood.

First, I have considered as, very generally, a demonstrable fact, that the malady is primarily ushered in by manifest derangement in those earlier actions of the process of nutrition by which the heterogeneous matters of aliment are converted into chyle.

Secondly, that the immediate influence of this error is directed to the blood, whose venous current becomes charged with unhealthy particles which the lungs fail to identify with the common mass in a due and proper manner.

Thirdly, that though this state of the blood exists, to a greater or less extent, in all cases of strumous habit, it does not necessarily lead to the actual location of tubercle either in the lungs or system.

Fourthly, that this condition of the blood forms one of the elements necessary to the production of the local affection, but that this requires for its full establishment, the presence of a peculiar structural condition of a portion of the capillary vessels connected with one or other of the circulations.

Fifthly, that the location of tubercles owns a mechanical cause, and depends on a change in the natural healthy relation which the particles of blood bear to the tubes through which it flows; and that tubercles are hence constituted by innumerable minute points contained within the calibre of obstructed vessels.

Sixthly, having in this manner attempted to trace the successive steps by which tubercular disease of the lung becomes established, I have approached the question of these phenomena which appear to depend on the re-action of lungs so circumstanced towards the system, endeavouring inferentially to show, that those are mainly dependant on an imperfect condition of the arterial blood, necessarily resulting, in many instances, from the existing condition of portions of the lung.

Seventhly, that this condition depends on a continuance of the pulmonic circulation through parts of the organ to which air no longer finds admission.

Whether these conclusions are warranted by the observations and reasonings contained in the preceding pages, must be determined by others than the author. I shall only here add, that as they were gradually arrived at by a careful examination of the malady, both in its vital and physical relations, so they have also appeared to afford a somewhat substantial foundation, on which to rest principles of treatment, whether directed to its alleviation or cure. To this portion of the subject it is now my duty to proceed." 217.

### TREATMENT.

At the fourth chapter the important—the most important, because tangible—portion of the work begins, with—"Is CONSUMPTION CURABLE?" We regret that our author did not, at once, refer to that noted "FELLOW" of Ely-place—that dignified member of our Profession—that eminent "BELLOWS-MENDER" of Holborn, who daily hints in the newspapers, that he can "stop holes in the lungs" with as much ease as his Brother Tinkers can mend the bottoms of saucepans. This would have saved a world of disquisition, the result only of which we shall here quote for the edification of the profession, and the consolation of the HECTIC.

"Passing over, therefore, for the present, any further allusion to the means by which consumption is most surely indicated, and assuming that the evidence of its existence in the cases subsequently stated was tolerably perfect, I shall proceed at once to consider the methods of treatment employed, and the principles on which this rests as directly connected with the general pathology of the disease set forth in the preceding pages; and I do so with the preliminary statement of my conviction, founded on no very limited experience, that *as a matter of fact, phthisis is not only frequently arrested in its progress, or remedied after full development by some obscure sanative effort of the system, but that, besides this, it is a disease amenable to the control of remedial means when these are applied in a proper manner, and directed under the guidance of a rational pathology.*" 221.

Whether the latter part of the passage, which we have marked in italics, correspond with the general experience of the medical world, we leave to our readers. It is rather at variance with our own experience.

The term *cure* is somewhat qualified by our author. By this phrase he does not mean to "imply the *entire* restoration of function and organization," but "such an approach to this as enables the individual, under certain limitations and modes of management, to carry on life without inconvenience, &c." We are quite ready to agree with Dr. Campbell, that Nature has given man such an ample apparatus for respiration, that a portion of lung may be rendered unfit for its ordinary functions, and yet the individual may go on performing the common duties of life for many years. We know hundreds of people who have considerable portions of lung hepatized—perhaps tuberculated—and only experience dyspnœa on making much exertion, with thinness and debility. If, therefore, we can devise the means of preventing the further deposit of tuberculous matter, before the lungs are too much charged with this destructive agent, we may prolong life, and even insure a comparative degree of health.

"The pathology of consumption, already sufficiently explained, recognises three stages:—

First, A primary stage, connected with erroneous action of the digestive organs, in consequence of which the matter of tubercle is presumed to be produced.

Secondly, A deteriorated state of the blood induced by this, during the continuance of which, that fluid presents one of the conditions necessary to the formation of tubercles, their location being contingent on a peculiar pulmonic organization, whose presence is therefore essential to a full production of the disease.

Thirdly, A stage of re-action towards the entire organs and functions of the body, as a direct result of tubercles after their location, the intensity of this being dependant not on their amount alone, but also on the peculiar influence which they frequently exert on the pulmonic circulation.

The indications of cure, which arise as direct corollaries out of these positions, are consequently three also:—

First, To counteract that morbid state of the digestive organs originating the matter, without which tubercles cannot be produced.

Secondly, To accomplish a solution of this matter, after it has passed into the blood, and thus arrest its local deposition, presumed to depend on mechanical retention in the extreme vessels of the pulmonary artery.

Thirdly, To place and retain the patient under such circumstances, in reference to his medical, dietetic, and general treatment, as seem on rational principles best calculated to meet those evils which result, from the existing state of his respiratory organs, thus affording time for removal by the natural process of softening of such tubercles as already actually exist." 225.

Our author acknowledges it as very improbable that we can remove the hereditary disposition to struma, and that we can only hope to counteract this tendency by improving the state of the digestive functions, and purifying the blood of its noxious ingredients. Dr. C. believes that strumous dyspepsia may exist without tuberculous deposition, but the latter never takes place without "strumous dyspepsia." Whether this be absolute or not, no harm can ensue from paying great attention to the said dyspepsia when it presents itself to our observation. It is characterized by two sets of symptoms—irritation of the mucous membrane of the bowels, and depraved secretions, especially of the liver. The papillæ of the tongue are red and prominent, often projecting through a dirty whitish fur, occupying the back and central surface, leaving the edges clean and bright red. The tongue itself is generally moist, flabby, and tremulous—sometimes dry and glazed, when the disease is intense. The tendency of the bowels is towards constipation, the evacuations being seldom healthy.

"The matter extruded is mixed with mucous or gelatinous discharges, sometimes tinged with blood, and various depraved secretions which render the stools frothy, green, and fætid; on other occasions the motions are of a colour considerably lighter than that of health, from an apparent suspension of the biliary secretion, while on others the liver acts with an augmented activity, of which a bilious diarrhœa is the result.

During this state of things, the abdomen is, almost constantly, tumid and drummy, the appetite capricious, though generally keen and craving, but nutrition languishes almost in proportion to the quantity of food which is received, and the lower extremities especially become weak and emaciated, sometimes appearing like slender dangling appendages to an inordinately bulky trunk." 229.

We need hardly remark that every medical practitioner is constantly in the habit of seeing daily such symptoms as the above in numerous cases, not one case in twenty of which leads to consumption, or indicates that disease, unless the fatal congenital or hereditary disposition or predisposition exists in the lungs themselves to receive these tuberculous deposits —or, where repeated attacks of inflammation in pulmonary structure itself have not occurred. This, indeed, is acknowledged by the author himself.

"The condition which they (the symptoms abovementioned) announce may become engendered, we have reason to think, even in a body of the most healthy structure, from various causes, such as bad or deficient food, impure air, and the like, especially during the earlier periods of life, but it is certainly more commonly found and more readily excited in those persons, stamped by what has been termed, a phthisical diathesis—a form of constitution undoubtedly transmitted downwards from parent to child, and often found to pervade the members of a whole family through many generations." 230.

We may safely pass over the signs of a phthisical diathesis as familiar to every student who walks an hospital. Although disordered conditions of the digestive organs and depraved secretions may doubtless accelerate the progress of tubercular deposition in the lungs—or even occasion that deposition, we firmly believe that another cause, or class of causes, is far more efficient, and far more frequent—we mean irritation or inflammation in the air-passages, induced by cold, by damp, by imperfect cloathing, and various atmospheric impressions, in consequence of which an afflux of blood, whether good or bad, is kept up in the lungs, and tubercular deposits accelerated or produced. While we attend to this portion of etiology, however, we have no objection to keep a strict eye on the one now under consideration—STRUMOUS DYSPEPSIA. Dr. Campbell's remarks on diet are judicious. In cases of this kind, it ought to be easy of digestion and containing nutriment of easy extraction by a disordered apparatus.

"Experience appears to determine that this is best accomplished by the employment of a mild farinaceous diet, which possesses the advantage of being converted into chyle, without producing that irritation of the digestive organs which more highly animalized articles but too generally excite. In milk we have presented to us an intermediate fluid, which from the very earliest ages has been extolled in the treatment of consumption in all its forms, and is not less adapted to its preliminary stage of which we now treat. There are very few cases in which it will not agree with the stomach, and should, in combination with the farinaceous food already named, form a large part of the diet employed." 235.

We are not prepared to go the whole length with our author on this point. The chief period of tubercular consumption lies between the age of fifteen and thirty-five, and when the digestive functions are much impaired, and the secretions vitiated. We shall find, in a majority of cases that milk will not agree, and, if the object be easy and complete digestion, plain animal food will answer better than milk. In respect to farinaceous food, it may be recommended where there is a tendency to inflammatory action in the respiratory apparatus, but it certainly is not the very best kind of nutriment for those who are disposed to strumous affections.

In the mean time we have morbid secretions to correct, local irritations to subdue, vigour to communicate to weakened organs of digestion, and many other indications to fulfil, not very easily done—the one often clashing with the other. In hepatic derangements, the author very properly condemns the frequent recourse which is had to calomel, when small doses of the pil. hyd. or the hyd. cum creta would be better. Thus a grain or two of the latter medicine with a little rhubarb and ginger, given every or every second night, till the colour of the secretions becomes natural, will be preferable to the chloride of mercury in most cases. When cough and irritation of the pulmonary apparatus shew themselves at an early period of the disease, our author places great dependence on ipecacuanha combined with the alteratives. As an aperient, castor oil is preferred by Dr. C. to most others. The omission of counter-irritation to the chest, or even of leeches, when cough actually exists, will be a very serious, and in many cases a fatal oversight. Dr. C. recommends the counter-irritation to be applied to the abdomen instead of the chest. We do not coincide with him, unless the irritation be solely confined to the gastro-intestinal mucous membrane. The warm bath is much recommended by our author. We would advise caution on this point—" hoc tu Romane caveto." On the subject of tonics we quite agree with our author.

"That the impaired action of the digestive organs depends, primarily, on some obscure changes in their vital relations, producing what may be termed debility in them, is at least probable; and that the general innutrition, or weakness which pervades the whole system, and frequently in turn establishes the local symptoms of struma in the lungs or elsewhere, arises directly out of this, is as certain as any proposition connected with vitality, but it by no means follows that the medicines generalized under the name of tonics, are those best adapted to such states. On the contrary the plain deduction from these premises appears to be, that the truly tonic plan of treatment depends on the adoption of means best calculated to rectify the original error, not by vain endeavours to obviate its effects; and hence chiefly depends on allaying irritation of the abdominal viscera, improving their secretions, and thus restoring them to that healthy habitude of action without which nutrition, and with it the vigour of the entire system, necessarily languish.

I feel called on therefore to express a strong conviction, that so long as the digestive tube remains in a congested irritable state, and so long as the abdominal secretions are improperly performed, the entire class of tonic and stimulating remedies always do harm, and that, to an extent proportioned to their strength. The case however is different when the abdominal symptoms have declined in whole or part. When the secretions have assumed an improved character, the tumidity of the abdomen subsided, and the tongue put on a more healthy aspect, then, and not till then, does it appear to me that a recourse to tonic remedies is at all admissible.

Even under such circumstances it is prudent to employ them cautiously. Infusions of the milder bitters should first be tried; if these produce no febrile excitement, they may either be used with more freedom, or an advance made to the various preparations of cinchona: iron seems to be only admissible when all symptoms of abdominal irritation have subsided; given under other circumstances, it usually aggravates the condition it is meant to cure." 248.

So much for "strumous dyspepsia," "a form of disease which may exist in full intensity, without being succeeded by tubercular deposition, but which, when it occurs in constitutions otherwise prone to take on that action, forms a most important element in its production."

But here we come to another therapeutical indication, dependent on the supposition that the blood is already more or less contaminated, and the morbid matter ready to be deposited, or beginning to be so, in the form of tubercles in the lungs. The nature of this contaminating principle is considered by our author as approaching nearly, if not exactly, to the "proximate principle, albumen," which may be detected in the blood of phthisical and cachectic invalids. The remedy or solvent for these nascent tubercles or their elements in the blood, is the caustic alkalis—a remedy employed for many years in the analogous malady, STRUMA itself. The solution of pure potash is the form used by our author, and great care is to be taken that it be not neutralised by any acid, even carbonic, before or after its entrance into the stomach. Where there are acidities in the primæ viæ, it should be combined with carbonate of potash or soda. We think the Doctor prescribes large doses of liq. potassæ, considering the great length of time that it is necessary to continue the medicine.

"With this view, children under the age of twelve or fourteen may take from fifteen to twenty-five drops of the liquor potassæ, three or four times in the day, according to circumstances, a quantity which with due perseverance is usually sufficient. In adults from a half to one drachm by measure, repeated at the same intervals, offers them whatever advantages the remedy is capable of producing." 257.

We have been in the habit of constantly prescribing this valuable medicine for more than thirty years past, but we confess that the above doses appear to us allopathic. Suppose an adult to take 240 minims daily (the maximum dose above) for weeks, months, or years. We apprehend that the mucous membrane of the stomach and bowels would become completely denuded of mucus, and that the muscles and fat would be pretty nearly absorbed. Our author recommends milk or distilled water as the vehicle for the alkali. Dr. C. candidly admits that neither this nor any other medicine "will prove effective in cases of consumption considerably advanced." But, as it is difficult to ascertain the degree of progress which the complaint has attained, a trial of the alkali may be made even in apparently desperate cases.

The 5th Chapter contains our author's views respecting the treatment of phthisis in its "STAGE OF REACTION." This stage occurs when the deposition of tubercles materially interferes with the respiratory and sanguifactive processes, and where the irritated or inflamed lung reacts on other organs or functions of the body. This chapter is thrown into three sections—diet—air, exercise, &c.—medicines. The most opposite advice has been given as to diet. One practitioner viewing consumption as a disease of pure debility, recommends beef-steaks and brown stout, as the best fare for a phthisical invalid; while another, looking to the inflammatory and febrile symptoms, enjoins the most rigid antiphlogistic regimen. Dr. C. endeavours to take a middle course, and avoid extremes. We know that animal food excites the action of the heart and lungs more than vegetable; but then, considering the imperfect condition of the digestive and respiratory apparatus, "we are often compelled to present to the digestive organs, articles containing as large an amount of nutritious elements as may be compatible with a due regard to the leading principle involved." A diet, therefore, moderately nutritious and sustaining, will generally be found the best. Milk, as before mentioned, Dr. C. considers as the head of the list of nutriments, and he seems convinced that it disagrees with people's stomachs much less frequently than is imagined, especially when combined with farinaceous matters. Then come the vegetable jellies, as Lichen Islandicus.

"Milk, well baked wheaten bread, and farinaceous aliments, in some one or other of the various forms which the art of cookery so liberally supplies, should thus constitute a large portion of the diet in all cases of phthisis, and the entire of it in those, where symptoms of associated inflammatory action prevail. When however this is not the case—where the acceleration of the pulse and respiration obviously depend on impeded action of the lungs alone—where the emaciation and debility are great—and where gastric irritation is not present to any marked extent—in such examples there seems a call for a nearer approach to that food, which in health is the most sustaining and nutritious." 276.

Then we are to have recourse to eggs, and fish once a day, or once in two days. When assimilative powers increase, without augmentation of excitement, we may allow a little game, poultry, soup, with farinaceous substances, beyond which Dr. C. thinks we should rarely go in the advanced stage under consideration. No better, he believes, can be laid down than this principle—"*that the use of a diet as highly nutritious as can be assimilated without leading to any exaggerated action of the breathing function, either directly or indirectly, is the one at all times most proper;* the moment this is exceeded we may feel well assured, that however sustaining the food employed may in itself be, aggravations will follow its adoption."

Wines and ardent spirits are prohibited, notwithstanding the complaints of debility and exhaustion so constantly made by patients. They stimulate, but offer no nutriment. Malt liquors come under a different category. "They may be considered as infusions of grain," containing a good deal of vegetable nutriment, "but associated with an amount of alcoholic stimulus, proportionate to their strength and richness." The brown stout, the Alton ale, and the Scotch malt liquors are hazardous drinks for the hectic lungs. Our author, however, admits them in quantities varying from half a pint to a pint in the day, "where there are no inflammatory symptoms—in short, in examples of purely chronic phthisis." But when do we meet with a disorganizing process going on in the lungs, without more or less of inflammation? We do not believe it possible. It is true that where a portion of lung is already broken down, and the patient is hastening to the tomb, we may, and perhaps ought to allow them those exhilarating malt liquors which give them even a temporary solace or support. But where we have any hope of arresting the progress of the malady, we believe that the bitter ales—Hodgson's, Bass's, or Richardson's, containing little malt and much hop, will be the best species of malt liquor for phthisical sufferers.

*Exercise, &c.*—The exaggerated opinion of Sydenham, who thought that "horse-exercise would as surely cure consumption, as bark an ague," has long been given up. *Active* exercise, indeed, is obviously injurious, as hurrying the circulation and embarrassing the lungs; but passive exercise in the carriage, or sailing, are beneficial, as tending to lessen the velocity of the pulse.

*Medicinal Agents.*—The medical world has been as much divided in respect to remedies as diet in consumption—some advocating stimulants and tonics—others antiphlogistics and depressants. We often see, however, that in the different stages of even the same case, both plans of treatment may be necessary. The chief question is, which plan is the most generally proper? Our author inclines to the class of *sedatives*, in preference to others, "as according best with the fulfilment of the object in view—the restriction, namely, of systemic action, with the ultimate view of diminishing the necessity for action by the lungs." *How* digitalis, prussic acid, and several other sedatives act, we are utterly ignorant. Nauseating medicines evidently reduce the force of the heart's action, and thus relieve the pulmonary apparatus; and it is remarkable that, from the earliest ages, emetics had the credit of curing more cases of phthisis than any other remedy. Of this class Dr. C. prefers the saturated solution of ipecacuanha in proof spirit;—because he generally combines it with the caustic alkali, and because the vinum ipecacuanhæ of the shops is often acid.

"Where narcotics are indicated, ipecacuan in substance may be advantageously combined, and the same may be said where slight mercurials or alteratives are demanded by the condition of the abdominal viscera. The quantity of the drug employed can scarcely be defined; much must depend on the circumstances of each case, and especially on the tolerance of the patient's stomach; the only general rule which can be laid down is, to excite a gentle nausea, frequently. To effect this purpose from ten to twenty drops of the saturated tincture generally suffices, and this may be repeated three or four times a day." 293.

Dr. C. doubts the propriety of exciting actual vomiting, under the supposition that the tuberculous matter lies on the surface of the mucous membrane, as maintained by Dr. Carswell, and may be dislodged by the act of vomiting. His own experience does not corroborate the views of Carson and Sir James Clark.

*Tonics.*—There is often (Dr. C. observes) an atonic condition of the digestive organs present, in which tonics are undoubtedly of use—but these organs must be free from marked signs of congestion or irritation.

"Of the various tonic remedies, the lighter bitters appear to me the best. Gentian, Calumba, Cascarilla, and many others, all offer valuable adjuncts to other and more effectual means; and in their infusions, present convenient vehicles for the administration of what I consider our great resource—the alkali. Care should be taken that the infusion employed has been recently prepared, as any evolution of free acid, the result of fermentation, renders it no longer a fitting vehicle for the associated remedy. From the Peruvian bark, or quinine, I have never seen any particular benefit derived." 298.

*Narcotics.*—The pure extract of lettuce, especially when combined with ipecacuanha, Dr. C. has found to be the best. But he considers the preparation, as found in the shops, completely useless. Dr. Duncan's form he thinks the only one that can be depended upon. The salts of morphia, and the liquor opii sedat. are the best forms of opiates.

In the second section of this chapter our author takes a leap or plunge —we hope not beyond his depth—"*can a function analogous to that of respiration be partially performed on other surfaces than those of the lungs?*" When we read this heading, we were painfully reminded of the vicarious senses of sight and hearing performed in the neighbourhood of Miss Okey's epigastrium. But the impression instantly vanished, for Dr. Campbell is anything but a mesmeric enthusiast. Experience has proved that certain substances received into the stomach reduce the temperature of the body, and along with this, diminish the force and velocity of the pulse. The explanation of this phenomenon is thus attempted by Dr. Murray, in his Elements of Chemistry.

"The animal temperature," says he, "is derived from the consumption of oxygen gas by respiration; and an increase in that consumption will occasion a greater evolution of caloric in the system, and consequently an increase of temperature; while a diminution in the consumption of oxygen will have an opposite effect. If then, when the temperature of the body is morbidly increased, we introduce into the stomach substances containing a large proportion of oxygen, especially in a loose state of combination, we may succeed in reducing the morbid heat."* 303.

There is no doubt but that all the remedies which experience has proved to be *refrigerants* contain much oxygen.

"We can scarcely fail to conclude, that even in a body structurally healthy, there exists a power of receiving and applying that element in such a manner, as to diminish the necessity of its reception by the lungs." 304.

If this be the case in healthy lungs, Dr. C. observes, it presents us a probable advantage where, from alteration of structure, the functional powers of extracting oxygen from the atmosphere are impaired. It is perhaps on this principle we can explain how acids, nitre and like remedies act. But it is evident that Dr. C., whose great object is to remove the tuberculous deposits by alkalis, cannot attribute much importance to acids and salines. In fact he objects to them, as interfering with the essential means of treatment. But by some experiments which Dr. C. made, it appears that when oxygen gas is kept for some time in contact with the skin, the blood circulating on the surface undergoes, to a certain extent, "changes similar to such as are effected on it in the pulmonary apparatus—in other words, prove that, within certain limits, the skin is to be viewed in man as a respiratory surface." If this be the case with respect to the skin, it is still more so as regards the mucous and serous tissues on the internal surfaces. Our author details some experiments on animals, where oxygen gas was injected into the intestines, and shewed the usual changes—absorption of a portion of the oxygen into the blood. In order that the cutaneous surface should be kept in the best state for absorption of oxygen, cleanliness and friction should be enjoined, and the air in contact with the skin should be frequently changed.

* Elements of Chemistry, Vol. I.

character, though the principle on which I presume their benefits to depend, is not the one commonly recognized." 321.

These steps taken, the next great object is, to change the air on the surface as often as possible. Riding on horseback or in a carriage, walking, sailing, swinging, &c., are to be adopted, according to the season of the year and the circumstances of the patient. Swinging and sailing are considered by our author as the best species of passive exercise.

*Inhalation.*—The third section of this chapter contains remarks on the inhalation of various medicinal substances, as the sedatives of opium, hyoscyamus, conium, prussic acid, &c., or of stimulants, as tar, chlorine, iodine. It is pretty clear, from the following extract, that Dr. C. does not rely much on inhalation.

"I have briefly noticed the principal remedies employed in consumption, as direct applications to the lung. That the method is sometimes useful cannot be questioned, but it should ever be considered as of secondary importance to the constitutional treatment we may deem proper; for, he who looks on phthisis as a local malady, and remediable by local means, will assuredly find himself mistaken. Neither is it to be forgotten, that if inhalation at times confers benefit, it may be productive of much evil; and that especially where stimulating vapours are employed, great care should be taken that little irritability of the pulmonary substance exists." 339.

The 4th section of this chapter contains cursory remarks on other points of local treatment, as adapted to certain symptoms, as inflammation, hectic fever, hæmoptysis, diarrhœa, colliquative perspiration, cough, &c. On these we need not dwell, as there is nothing very different in these remarks from the opinions and practice of the experienced portion of the profession.

The last chapter is occupied with a statement of cases, preceded by a short, but very valuable dissertation on the diagnosis of tubercles in the lungs. To ascertain the existence of these bodies in their early stages of deposition and development is extremely difficult. The attempt to rely on auscultation and percussion *alone*, in such cases, is highly preposterous. We need and require the aid of symptomatology, history, hereditary tendencies, &c., and we shall often derive more authentic information from these, in incipient cases, than from physical signs.

"Percussion and auscultation are without doubt the best and most certain means of diagnosis, which phthisis admits; and were we limited to one set of marks alone, we should scarcely hesitate to adopt them in preference to all others. They are not however infallible—and I hold that in the very early stages of the disease, we are scarcely justified in concluding fiom them only, that tubercles are present, unless the evidence they offer is also corroborated by the history of the case—the hereditary tendencies of the patient, and the nature of the symptoms which are present." 368.

The principal signs on which auscultators have relied are, greater or less dulness of sound on percussing the upper portions of the chest—absence or diminution of the respiratory murmur in the same situation—and resonance of the voice or cough. These being all distinct, consolidation of the lung is pretty certain—and if in the upper lobes, the great probability is that the solidification results from tubercles—especially if the patient evince the usual phenomena of phthisical tendency. But then it is by no means always that such physical signs are all present or unequivocal. In the early stages of the disease they are very much wanting. Yet even here, the experienced eye will pretty generally detect the lurking and mortal malady through the medium of other signs than auscultatory.

"The general aspect of the patient—almost intuitively recognized by those accustomed to observe it; the panting respiration—the quick irritable pulse, inordinately accelerated on the least exertion—the usual presence of struma in some external organ—and the contracted, flat contour of the chest, present a combination of symptoms which it is all but impossible to mistake; and we shall seldom find that these are not amply confirmed, by a recourse to auscultation and percussion. It is not however always that symptoms are so distinct, and without question, in numerous examples the general signs amount to suspicion only, while the physical ones, either render these more conclusive, or at times announce tubercular occupation with precision, where the others are too indistinct, to attract much attention or lead to much alarm. It is therefore especially proper that we should duly consider their relative value, and estimate properly the circumstances under which they may without deception be relied on." 369.

Our author attaches more importance to percussion than to auscultation in the early stages of tubercular deposition; for when tubercles are scattered through the lungs, and at some distance from each other, the respiratory murmur in the intervening spaces can only be pronounced abnormal by those whose ears are very long accustomed to the stethoscope. Both auscultation and percussion, however, ought always to be conjoined, and neither of them trusted to singly.

Our author details ten cases (chiefly dispensary patients) where the treatment appeared to have conduced to the quiescence of existing pulmonary tubercles—three tending to shew the possibility of tubercular absorption—and three cases of phthisis in its very advanced stage benefitted by the alkali. These cases appear to be very honestly and candidly stated; and as they must all have been seen by others at the public institution where they occurred, they possess a degree of authenticity, which can seldom be claimed by the histories of cases in private practice. These narratives we must leave to the careful perusal of our readers, as we have considerably exceeded the bounds which we first laid down for analysis of this work. The interest of Dr. Campbell's book regularly increased as we proceeded in the perusal of it, and we conscientiously believe that it is the best and most *practical* treatise on the subject that has appeared in the English language. The style is clear and unadorned, but terse and classical. It is perfectly free from the slightest tincture of quackery, mystery, or puffery; and a tone of candour, modesty, and veracity pervades every page.

